# Direct Experimental Demonstration of Bend-Induced Transformation of Magnetic Structure in Amorphous Microwires

**DOI:** 10.3390/s25165000

**Published:** 2025-08-12

**Authors:** Alexander Chizhik, Valentina Zhukova, Arcady Zhukov

**Affiliations:** 1 Department Advanced Polymers and Materials: Physics, Chemistry and Technology, University of Basque Country, UPV/EHU, 20018 San Sebastian, Spain; valentina.zhukova@ehu.eus; 2 Department of Applied Physics, University of Basque Country, UPV/EHU, 20018 San Sebastian, Spain; arkadi.joukov@ehu.eus; 3 IKERBASQUE, Basque Foundation for Science, 48011 Bilbao, Spain

**Keywords:** amorphous magnetic microwires, magneto-optic Kerr effect, bending stress, bending sensors

## Abstract

In the pursuit of active elements for bending and curvature sensors, magneto-optical investigations were performed on bent microwires. For the first time, local surface magnetization reversal curves were obtained from various sides of bent Co-rich and Fe-rich microwires. The observed differences in surface magnetization reversal behavior are directly attributed to the transverse distribution of internal mechanical stresses, which range from maximum tensile stress on the outer side of the bent sample to maximum compressive stress on the inner side. Depending on the sample composition and the nature of local stress, distinct magnetic structures—axial, elliptical, and spiral—were identified in different locations on the surface of the microwire. These findings provide valuable insights into the operational mechanisms of bending-sensitive magnetic sensors.

## 1. Introduction

The impact of mechanical bending on the physical properties of elongated samples has attracted scientific attention since the early 20th century [1,2,3,4,5,6,7]. This interest is driven by both the fundamental physical challenges associated with bending and its practical relevance to sensor technologies [8,9,10,11,12,13,14,15]. In this context, our research specifically explores how mechanical bending influences the magnetic and electrical properties of materials.

Two primary factors motivate our current investigation: the emerging field of curvilinear magnetism [12], and recent advancements demonstrating the use of magnetic wires as active components in functional bending sensors [6]. These motivations are closely intertwined and collectively define the scope of our work. Glass-coated magnetic microwires have become a prominent subject in contemporary physics due to their unique magnetic behavior. They exhibit a variety of magnetic domain structures—axial, circular, and spiral—both internally and on their surfaces, making them ideal candidates for studying the effects of curvature on magnetic phenomena. We also find this topic important in the context of general research into magnetic bending sensors and high-frequency sensors [16,17].

Magnetic wires exhibit remarkable magnetic properties, most notably the giant magnetoimpedance (GMI) effect and magnetic bistability [18,19]. These properties are intrinsically linked to the distinctive domain structure of such wires, which typically consists of an axially magnetized inner core enveloped by an outer domain shell [20,21]. The strong GMI effect is attributed to the high circumferential magnetic permeability [22,23], while magnetic bistability results from the rapid propagation of a single domain wall (DW) within the inner core.

The emerging field of curvilinear magnetism encompasses a wide range of length scales, from the nanoscale to the millimeter scale. However, the current literature indicates that the micrometer scale remains relatively underexplored. Our preliminary studies in this domain have demonstrated that flexible, elastic microwires are well-suited for investigating the effects of mechanical bending on magnetic behavior. These materials produce a broad spectrum of experimental outcomes, many of which offer novel and insightful contributions to the understanding of curvature-induced magnetic phenomena.

The primary technique employed in our research is the magneto-optical Kerr effect (MOKE), with MOKE magnetometry serving as the principal method for investigating the magnetic structure at the surface of microwires. This approach provides detailed insights into the surface magnetization reversal process and enables us to monitor the influence of various external parameters, including combinations of magnetic fields, mechanical stress, temperature, and others.

In earlier studies focused specifically on the effects of bending stress, we adopted the following experimental approach [24]: microwire samples were pre-bent—sometimes shaped into geometric spirals—and subsequently annealed in a furnace. After annealing, the samples were returned to their original straight configuration for analysis. Remarkably, the magnetic structure modifications induced by bending were retained in the annealed samples. These preserved changes were then characterized, leading to the key conclusion that a transverse stress gradient is established across the microwire’s axial cross-section as a result of bending and thermal treatment.

In the present study, we adopt a different experimental approach: more complex in execution but more representative of practical applications in real bending sensors. A long microwire sample was bent and investigated using the MOKE technique while remaining in its bent configuration. Acquiring reliable optical reflections from a bent cylindrical surface posed certain experimental challenges, which were successfully addressed. Our goal was to obtain magnetic field-dependent surface magnetization reversal curves from various localized regions of the bent sample, enabling a longitudinally spatially resolved analysis of the magnetic behavior under mechanical deformation.

## 2. Experimental Details

We investigated glass-coated microwires with the following chemical compositions: Co_64.04_Fe_5.71_B_15.88_Si_10.94_Cr_3.4_Ni_0.03_ (Sample 1), with metallic nucleus diameter d = 94.6 μm and total diameter with glass covering D = 126 μm; and Fe_71.8_B_13.27_Si_11.02_Nb_2.99_Ni_0.92_ (Sample 2), with metallic nucleus diameter d = 47.5 μm and total diameter with glass covering D = 57.6 μm. Both samples were fabricated using the Taylor–Ulitovsky technique. The length of each sample was approximately 10 cm.

The surface magnetization reversal process of the microwire was investigated using a magneto-optical Kerr effect (MOKE) loop tracer system [25]. Polarized light from a He–Ne laser was directed onto the microwire, and the reflected signal was subsequently detected. The laser spot size was about 1 mm in diameter. The angle of light incidence was around 40° relative to the microwire axis. The incident light was linearly polarized (s-polarization). The wire was fixed onto a holder so that the light is reflected onto the detector from a part of the microwire about 1 mm long. In the longitudinal MOKE configuration, the rotation of the polarization angle of the reflected light is directly proportional to the magnetization component parallel to the plane of incidence.

Due to the cylindrical geometry of the wire, the reflected light forms a conical surface, which can introduce distortions in the magneto-optical signal. To minimize these distortions, an optical setup incorporating an aperture diaphragm and a system of lenses was used to select the light reflected from a defined, localized section of the microwire’s surface. This configuration allowed for accurate and longitudinally spatially resolved measurement of surface magnetization behavior.

**Figure 1:**
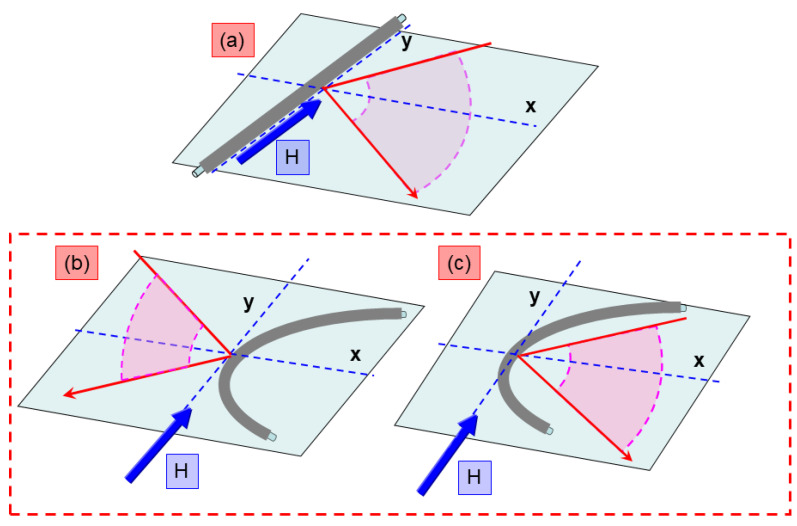
Schematic pictures of experiments. (**a**) MOKE experiment with unbent sample; (**b**) MOKE experiment with light reflection from outer surface; (**c**) MOKE experiment with light reflection from inner surface. The dashed red line highlights original MOKE configurations.

The experimental procedure is outlined in Figure 1. Initially, MOKE hysteresis loops were recorded from a straight, unbent microwire sample (Figure 1a). The sample was then bent (the bending radius applied to samples was 7 cm), and MOKE measurements were taken from both sides of the curved wire, which, for clarity, are referred to as the “outer” side (Figure 1b) and the “inner” side (Figure 1c). For the straight sample (Figure 1a), the external magnetic field generated by Helmholtz coils was applied along the longitudinal axis of the microwire. In the case of the bent sample (Figure 1b,c), the magnetic field was oriented parallel to the local longitudinal axis of the microwire local segment from which the laser light was reflected, ensuring consistent magnetic field alignment with the plane of the light.

## 3. Results and Discussion

**Figure 2:**
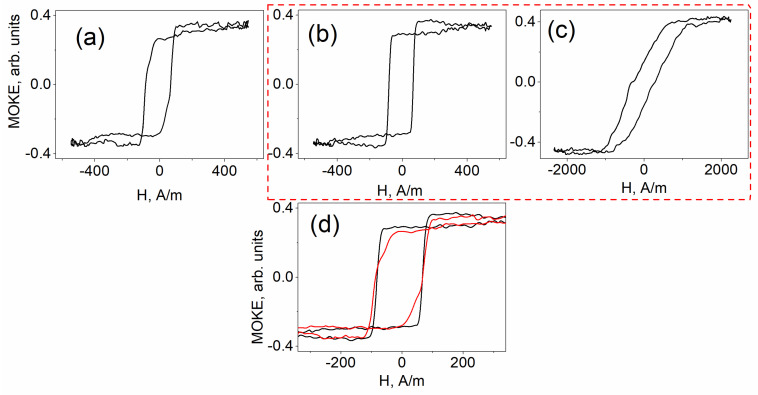
MOKE hysteresis loops obtained from the surface of Co-rich microwire. (**a**) Reflection from unbent sample; (**b**) reflection from outer side; (**c**) reflection from inner side; (**d**) comparison of MOKE hysteresis presented in (**a**,**b**). The dashed red line highlights the MOKE response obtained from the curved sample, an experimental result reported here for the first time.

**Figure 3:**
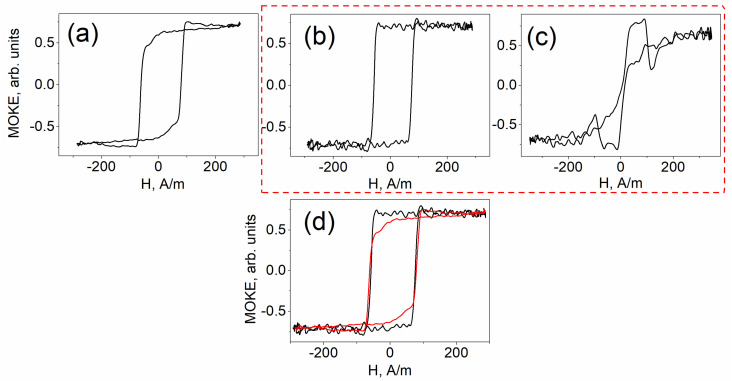
MOKE hysteresis loops obtained from the surface of Fe-rich microwire. (**a**) Reflection from unbent sample; (**b**) reflection from outer side; (**c**) reflection from inner side; (**d**) comparison of MOKE hysteresis presented in (**a**,**b**); The dashed red line highlights the MOKE response obtained from the curved sample, an experimental result reported here for the first time.

Figure 2 and Figure 3 present the results of MOKE measurements for the Co-rich and Fe-rich microwire samples, respectively. In both cases, Figure 2a and Figure 3a display the hysteresis loops obtained from the straight, unbent samples. Figure 2b and Figure 3b show the MOKE hysteresis curves recorded from the outer side of the bent samples, while Figure 2c and Figure 3c correspond to measurements from the inner side. For comparison, Figure 2d and Figure 3d overlay the hysteresis loops from the unbent (a) and outer-side (b) measurements. The dashed red line highlights the MOKE response obtained from the curved sample, an experimental result reported here for the first time.

MOKE hysteresis behavior similar to that shown in Figure 2a was previously observed in our earlier studies on Co-rich microwires [26]. This type of hysteresis loop is typically associated with the motion of elliptical domain walls, followed by gradual magnetization rotation toward the axial direction. Figure 2b clearly reveals the manifestation of axial magnetic bistability, characterized by the abrupt propagation of a single domain wall along the length of the sample. In contrast, the MOKE response obtained from the inner surface of the bent sample (Figure 2c) exhibits a smooth, continuous variation in the signal, lacking the sharp transitions indicative of domain wall jumps. Figure 2d offers a direct comparison, illustrating how the smooth behavior related to elliptical domain wall motion (red line) transitions into discrete jumps between axial domains (black line) under the influence of external bending stress.

A corresponding set of MOKE hysteresis curves was obtained for the Fe-rich microwire sample, as shown in Figure 3. Figure 3a presents the surface magnetization reversal curve for the unbent sample. Figure 3b and Figure 3c illustrate the modifications to the hysteresis loops induced by bending, with measurements taken from the outer (Figure 3b) and inner (Figure 3c) surfaces, respectively. Figure 3d provides a direct comparison between the hysteresis curve recorded prior to bending (red line) and that obtained after the application of bending stress (black line), highlighting the influence of mechanical deformation on the magnetization behavior.

What are the key features observed in the case of Fe-rich microwires? In the absence of external mechanical deformation (Figure 3a), the magnetization reversal curve exhibits a sharp jump, followed by a gradual rotation of the magnetization toward the axial direction. This behavior has previously been identified as elliptical bistability, attributed to the rapid propagation of a single elliptical domain wall [27]. When measured from the outer surface of the bent sample (Figure 3b), this elliptical bistability transitions into axial bistability. A direct visual comparison of these two regimes is presented in Figure 3d. It is evident that the magnetization jump in the unbent sample is smaller than the jump observed from the curved outer surface, highlighting the transformation induced by bending. This comparison clearly demonstrates the manifestation of elliptical bistability in the straight (unbent) microwire.

The most unexpected result in this series is shown in Figure 3c. The magnetization reversal curve exhibits a sequence of localized peaks and troughs in the MOKE signal. Initially surprising, this behavior closely resembles the surface magnetization reversal response previously observed in microwires of identical composition subjected to longitudinal torsional stress. As established in our earlier work [28], such a pattern of changes in the MOKE hysteresis loop is indicative of the formation, evolution, and propagation of a helical or spiral magnetic structure along the wire’s surface. The similarity of this characteristic response in the current experiment suggests that a similar helical configuration is present under the applied bending conditions.

As a preliminary analysis, it is important to consider the following observations, which help explain the apparent similarity in magnetization reversal behavior observed under two seemingly different experimental conditions: the application of torsional stress [26] and bending stress (this study). It has been shown that, in a long cylindrical rod subjected to torsional loading, the resulting internal stress distribution can be described as a superposition of tensile and compressive components [29]. Specifically, torsional stress induces a combination of tensile and compressive stresses oriented obliquely relative to the rod’s axis. The maximum tensile stress is directed at approximately +45° to the axis of applied torque, while the maximum compressive stress occurs at −45°. This angular distribution of internal stresses provides a common framework for interpreting the similar magnetic responses observed in both torsion- and bending-induced transformation of magnetization reversal processes.

Uniquely, in both cases—torsional and bending stress—the external mechanical loading includes a compressive stress component. This type of stress may play a key role in the formation of spiral magnetic structures. One important preliminary conclusion, drawn directly from the experimental results without detailed analysis of internal stress distribution, is as follows: this study provides the first experimental evidence that, in a mechanically bent microwire, the magnetization reversal process proceeds in fundamentally different ways on opposite surfaces, i.e., the inner and outer sides. This finding was made possible by the localized sensitivity of the MOKE technique, which allows longitudinally spatially resolved observation of regions subjected to varying magnitudes and directions of mechanical stress.

The relationship between mechanical stress applied to an elongated sample and the resulting curvature was first established in a seminal work [1], which examined the effects of bending stress on the properties of metallic films. This foundational concept was later expanded to encompass a broader range of materials and experimental approaches [2,3]. A notable contribution marking the centenary of Gerald Stoney’s formula [3] highlighted its continued relevance and introduced modifications to account for layered structures subjected to bending.

In the early stages of our analysis, we found particularly valuable a study that mathematically described the distribution of bending stress in a long cylindrical sample from a purely mechanical perspective, introducing the concept of circular cross-sections [30]. A critical advancement came with the application of this mechanical framework to magnetic systems. Specifically, the established connection between bending, magnetostriction, and anisotropy fields provided a foundational understanding of how bending influences the magnetic properties of curved magnetic materials [4].

The most relevant study for our work was one in which a modified version of Stoney’s formula was applied to magnetic microwires utilized in sensor applications [6]. The successful demonstration of a functional bending sensor based on a microwire underscored the importance of employing localized magneto-optical analysis in such systems.

**Figure 4:**
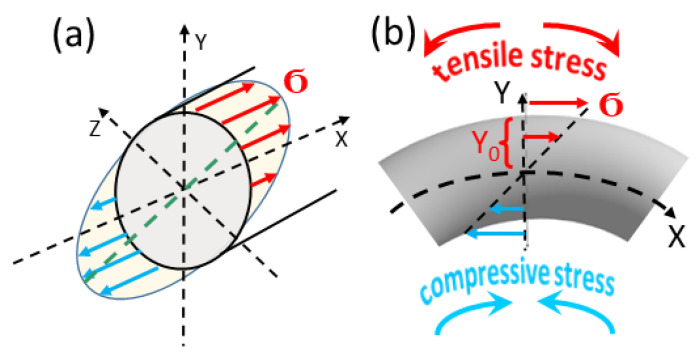
Distribution of bending stress in volume of sample (**a**) and in cross-section (**b**). Red and blue arrows show the direction of tensile and compressive stress, respectively. The stress value changes both in the sample volume and on the sample surface. In the case of spiral annealing, the absolute value of the induced stress decreases with increasing spiral radius (decreasing curvature).

Accordingly, in our interpretation of the experimental results, we adopted the following framework: the correlation between externally applied bending stress and the resulting curvature of an elongated sample formed the basis of our analysis. Furthermore, we assumed that a bent microwire exhibits a transverse spatial distribution of internal mechanical stress across its cross-section, as schematically illustrated in Figure 4.

It is now understood that bending stress in a microwire consists of tensile stress distributed along the outer region and compressive stress along the inner region, with zero stress occurring along the neutral axis, as illustrated in Figure 4. According to Stoney’s formula [1,2], which first established the relationship between mechanical stress and the curvature of a bent structure, the magnitude of the stress σ acting across the wire’s cross-section can be calculated using the following expression:

σ = −E∙Y/r = −E∙Y∙C(1)

In this expression, E represents the Young’s modulus of the microwire, Y is the distance from the neutral axis, r is the radius of curvature, and C denotes the curvature. According to the formula, the stress σ is negative when Y is positive—corresponding to the outer (tensile) region of the microwire—and positive when Y is negative—corresponding to the inner (compressive) region. As a result, the overall strain across the wire’s cross-section is theoretically balanced, with equal magnitudes of tensile and compressive stress. However, detailed mechanical modeling [30] and experimental studies involving magnetic impedance measurements [6] have demonstrated that the effects of tensile and compressive stress are not perfectly symmetrical. Consequently, tensile stress tends to exert a more pronounced influence on the magnetic behavior of bent microwires.

The relation between applied mechanical stress and magnetic properties is described by the well-known expression for the induced change in the anisotropy field [31]:

H_k_ = 3λσ/M_S_ (2)

In this equation, σ denotes the applied stress, λ is the saturation magnetostriction constant, and M_S_ is the saturation magnetization. This formula establishes the direct link between applied mechanical stress and the resulting changes in the magnetic anisotropy in the material.

By combining Equations (1) and (2), a direct relationship is established between the magnetic properties of the microwire and the nature of the externally applied mechanical stress. Of particular relevance to our MOKE investigations is the transverse variation of stress as a function of the coordinate Y (as shown in Figure 4), which transitions from tensile to compressive across the wire’s cross-section. Specifically, at Y = +Y_0_ (Figure 4b), the tensile stress reaches its maximum, while at Y = −Y_0_, the compressive stress is at its peak. This spatial variation in stress distribution manifests in our localized MOKE measurements, providing clear experimental evidence of its influence on magnetization behavior.

To calculate the absolute value of stress on the upper and lower surfaces of the two samples, we used the following constants. Sample 1: Young’s modulus E = 190 GPa, sample radius Y_0_ = 47.3 μm, bending radius r = 0.07 cm; Sample 2: Young’s modulus E = 160 GPa, sample radius Y_0_ = 27.8 μm, bending radius r = 0.07 cm.

As a result, we obtained the absolute values of the stress as 128 MPa (Sample 1) and 63 MPa (Sample 2), respectively. The values corresponding to the tensile stress fall within the usual range of applied tensile stresses that we use in our experiments. The tension-induced transformations of the MOKE hysteresis curves correspond to the expected changes. As for the effect of compression, we can say that the intermediate stress values cause significant changes in the MOKE hysteresis. Since this is the first study of the effect of compression on the magnetic structure of microwires, this result requires further research.

Based on our findings, the following picture emerges regarding the transformation of the magnetic structure in the studied Co-rich and Fe-rich microwires. Both samples, characterized by their long cylindrical geometry, develop a transverse gradient of mechanical stress under applied bending. This stress distribution varies continuously from maximum tensile stress at the outer surface to maximum compressive stress at the inner surface. Prior to the application of bending, both microwires exhibited a uniform elliptical domain structure distributed consistently along both the axial and radial directions. Distinct magnetization behaviors were observed in the two compositions: in the Co-rich sample, the elliptical domain walls exhibited relatively smooth and continuous motion, whereas in the Fe-rich sample, a characteristic elliptical magnetic bistability effect was detected.

The application of bending stress leads to the emergence of the axial magnetic bistability on the outer surfaces of both samples, characterized by the propagation of a single, compact domain wall (Figure 2b and Figure 3b). This behavior is characteristic of microwires exhibiting positive magnetostriction.

In contrast, the magnetization reversal process on the inner surface differs markedly. This region experiences maximum localized compressive stress, a condition that cannot be replicated by other known methods of mechanical loading. For example, under torsional stress, tensile and compressive components coexist and overlap [29]. On the inner surface of the Co-rich sample, the MOKE hysteresis response is smooth and continuous, without abrupt signal changes, indicating a gradual rotation of the magnetization rather than domain wall formation. This behavior is characteristic of microwires with negative magnetostriction.

On the inner surface of the Fe-rich sample, the hysteresis response exhibited distinct dips and abrupt jumps. Based on our previous observations [28], this field-dependent behavior is attributed to the magnetization reversal of a spiral magnetic structure that is likewise characteristic of microwires with negative magnetostriction.

Within the framework of the present analysis, an open question persists regarding the mechanism of the magnetic structure transversal transition between the distinctly different configurations observed on the upper and lower surfaces of the microwire. Addressing this issue will require more advanced and integrated experimental approaches. It is worth noting, however, that the simultaneous coexistence of different magnetic structures has previously been observed in our studies and was associated with the longitudinal variation of magnetic properties along the sample. Additionally, we have documented the formation of distinct magnetic structures depending on the direction of the applied magnetic field.

A critical area for further investigation is the potential application of the studied microwires in magnetic field bending sensors based on the GMI effect. It is well established that transverse magnetic susceptibility plays a pivotal role in understanding and optimizing the GMI response. In this context, the surface magnetic domain structure contributes significantly to the overall transverse susceptibility. Specifically, the presence of a well-defined domain structure is known to enhance transverse susceptibility at low operating frequencies, which is advantageous for sensor performance in this range. Conversely, at higher frequencies, a smooth and continuous magnetization reversal becomes more influential in determining the GMI response.

As demonstrated in present experiments, the Co-rich sample exhibits predominantly smooth magnetization rotation under compressive stress (Figure 2c), whereas the Fe-rich sample, under identical external conditions, develops a spiral domain structure (Figure 3c). These observations suggest that Co-rich microwires are more suitable for high-frequency applications, where continuous magnetization rotation enhances performance, while Fe-rich microwires may be more effective at low frequencies, where the presence of a domain structure contributes significantly to transverse susceptibility.

Compressive stress in magnetic microwires, particularly those with positive magnetostriction, can lead to the formation of spiral domain structures through stress-induced helical anisotropy. This mechanism arises from the magnetoelastic coupling between the internal stress field and the local magnetization, favoring a spiral alignment that balances mechanical, magnetic, and geometric constraints. The resulting domain structure is a low-energy configuration that reflects the internal stress gradient and the cylindrical symmetry of the system.

Thus, for materials with positive magnetostriction (λ > 0), such as Fe-rich microwires, compressive stress induces a negative magnetoelastic anisotropy, which energetically favors magnetization directions perpendicular to the stress axis. In cylindrical geometries, this translates to spiral (in particular cases, circumferential) magnetic alignment.

In contrast, for materials with negative or near-zero magnetostriction (λ ≤ 0), such as Co-rich microwires, the same compressive stress either generates a weaker anisotropic response or promotes magnetization alignment along the direction of the stress, resulting in axial uniform magnetic configurations.

Consequently, under compressive stress, Fe-rich microwires tend to develop spiral or circumferential magnetization. Co-rich microwires exhibit axial alignment and smooth coherent magnetization rotation, rather than forming well-defined domain structures.

Regarding the possible use of microwires in bending sensors, the following can be said. Our proposal regarding the high-frequency applicability of bent microwires is supported by recent advances in the field demonstrating the sensitivity of the microwave response to mechanical stress in such systems.

In particular, it has been shown that Co-rich amorphous microwires, due to their negative or near-zero magnetostriction, exhibit a smooth magnetization rotation under compressive stress, as confirmed in our MOKE experiments. This behavior is highly advantageous at microwave frequencies, where low-loss, continuous magnetization dynamics is desirable. The following studies directly support this.

In [17], it was demonstrated that multilayer metal spin-valve nanostructures exhibit high magnetoresistance at ultra-high frequencies up to 40 GHz. This highlights the suitability of layered structures for microwave field sensing in the GHz range. In [6], the microwave impedance spectroscopy of glass-coated amorphous wires was used to detect local bending stress, showing resonant frequency shifts and GMI changes under stress in Co-rich wires, reinforcing their potential as active elements in bend-dependent wireless sensors. Most recently, in [32], it was experimentally shown that free-space microwave measurements on Co-rich amorphous microwires enable remote stress monitoring, with the stress-induced anisotropy directly influencing the high-frequency response.

These findings are consistent with our interpretation: bending stress in Co-rich wires induces a transverse anisotropy that promotes uniform rotation rather than domain wall motion, resulting in a low-noise, high-speed magnetic response favorable for GHz-range applications. In contrast, Fe-rich wires, with positive magnetostriction, tend to form spiral or bistable domain structures under bending stress, leading to more abrupt, non-linear magnetization dynamics. This makes them more suitable for low-frequency or switching-based sensing.

## 4. Conclusions

Local surface magnetization reversal in two types of magnetic microwires was investigated using the MOKE technique under applied bending stress. For the first time, a pronounced difference in the magnetization reversal behavior between the outer and inner surfaces of bent cylindrical samples was observed, as evidenced by distinct MOKE hysteresis loop characteristics. Additionally, the magnetization reversal features were found to be dependent on the type of magnetic microwire. Furthermore, a clear distinction was observed between the magnetization reversal processes in bent and unbent samples, highlighting the influence of mechanical deformation on magnetic behavior.

The interpretation of the magnetic structure in bent magnetic microwires is based on a model that incorporates a transverse gradient of internal stress induced by bending. In this model, the outer surface of the wire experiences maximum tensile stress, while the inner surface is subjected to maximum compressive stress. The magnitude of this stress gradually decreases with depth into the material, with a transition line where tensile stress gives way to compressive stress. Based on this framework, our MOKE results show that tensile stress locally induces axial magnetic bistability in both types of microwires. In contrast, compressive stress can lead to the development of either a spiral domain magnetic structure or a homogeneous magnetic configuration characterized by transverse anisotropy. In the absence of bending, the magnetic structure in both samples is predominantly elliptical.

The external bending-induced selection of magnetic structure offers valuable insights for selecting the appropriate type of microwire as an active element in bending sensors designed to operate across different frequency ranges. This dependent behavior under mechanical bending stress can thus serve as a criterion for optimizing sensor performance based on application-specific frequency requirements.

## Data Availability

Data available on request due to restrictions related to the developing.
